# Morin, a Bioflavonoid Suppresses Monosodium Urate Crystal-Induced Inflammatory Immune Response in RAW 264.7 Macrophages through the Inhibition of Inflammatory Mediators, Intracellular ROS Levels and NF-κB Activation

**DOI:** 10.1371/journal.pone.0145093

**Published:** 2015-12-28

**Authors:** Chitra Dhanasekar, Sowmiya Kalaiselvan, Mahaboobkhan Rasool

**Affiliations:** Immunopathology Lab, School of Biosciences and Technology, VIT University, Vellore-632014, Tamil Nadu, India; Queen Mary University of London, UNITED KINGDOM

## Abstract

Our previous studies had reported that morin, a bioflavanoid exhibited potent anti-inflammatory effect against adjuvant-induced arthritic rats. In this current study, we investigated the anti-inflammatory mechanism of morin against monosodium urate crystal (MSU)-induced inflammation in RAW 264.7 macrophage cells, an *in vitro* model for acute gouty arthritis. For comparison purpose, colchicine was used as a reference drug. We have observed that morin (100–300 μM) treatment significantly suppressed the levels of inflammatory cytokines (TNF-α, IL-1β, IL-6, MCP-1 and VEGF), inflammatory mediators (NO and PEG_2_), and lysosomal enzymes (acid phosphatase, β-galactosidase, N-acetyl glucosamindase and cathepsin D) in MSU-crystals stimulated macrophage cells. The mRNA expression of pro-inflammatory cytokines (TNF-α, IL-1β, IL-6, and MCP-1), inflammatory enzymes (iNOS and COX-2), and NF-κBp65 was found downregulated in MSU crystal stimulated macrophage cells by morin treatment, however, the mRNA expression of hypoxanthine phospho ribosyl transferse (HPRT) was found to be increased. The flow cytometry analysis revealed that morin treatment decreased intracellular reactive oxygen species levels in MSU crystal stimulated macrophage cells. The western blot analysis clearly showed that morin mainly exerts its anti-inflammatory effects by inhibiting the MSU crystal-induced COX-2 and TNF-α protein expression through the inactivation of NF-κB signaling pathway in RAW 264.7 macrophage cells similar to that of BAY 11–7082 (IκB kinase inhibitor). Our results collectively suggest that morin can be a potential therapeutic agent for inflammatory disorders like acute gouty arthritis.

## Introduction

Gouty arthritis is the most painful arthritis caused by an inflammatory reaction that arises in response to the deposition of uric acid in the form of monosodium urate (MSU) crystals in articular joints and bursal tissues of individuals with hyperuricemia, provoking robust inflammation and unbearable pain [1,2]. Epidemiological evidence suggests that in developed countries, 1% of the population are affected with gouty arthritis with most common occurrence among men and post-menopausal women. Its incidence and prevalence increase significantly in the individuals who live an unhealthy lifestyle and consume thiazide diuretics, prophylactic aspirin, and alcohol frequently [3]. Uric acid is a catabolite of purine metabolism that is produced in high quantities upon cellular injury. Uric acid released from injured cells forms MSU crystals upon binding by uric-acid specific antibodies. A preponderance of literature suggests that MSU crystals can be recognized as an endogenous adjuvant and pro-inflammatory signals analogous to a motif, called danger associated molecular pattern (DAMP) by innate phagocytes including dendritic cells, macrophages and neutrophils. These DAMPs that are similar to pathogen-associated molecular pattern can drive systemic inflammatory immune responses in the absence of infectious triggers [4]. Several investigators have demonstrated that the initial process of inflammatory response occurs when articular resident macrophages that are present within the joint space phagocytose MSU crystals. Significantly, MSU crystals that have been engulfed by macrophages interacts with pathogen-recognition receptors, Toll-like receptors (TLR) 2/4 and CD 14 likely triggers the MyD88/TRIF pathway that leads to nuclear factor-κB (NF-κB) activation and formation of a protein complex called NLRP3 inflammasome, resulting in the activation of caspase-1 and processing and secretion of IL-1β, a pro-inflammatory cytokine. IL-1β along with other pro-inflammatory cytokines, TNF-α, IL-6, and IL-8 promote neutrophil influx, the primary pathological hallmark of gout [5]. Infiltrating neutrophils exert their detrimental role at the inflamed joints, mainly through the extracellular release of variety of mediators, including reactive oxygen species, proteolytic enzymes, cytokines, chemokines and prostaglandin E_2_ (PGE_2_) that ultimately progresses to cartilage degradation and joint damage [6,7]. A study by Martin et al [8] reported that resident macrophage depletion significantly inhibited neutrophil infiltration at the inflamed joints and abrogated the production of pro-inflammatory cytokines, including IL-1β, suggesting that resident macrophages are key in initiating the inflammatory cascade. It has been speculated that the inhibition of the formation of these inflammatory mediators and/or the NF-κB signaling pathway in macrophages could serve as a useful therapeutic approach to treat acute gouty arthritis. Acute gouty arthritis is usually managed by the administration of oral colchicine, non-steroidal anti-inflammatory drugs (NSAIDs), and glucocorticoids. The development of therapeutics targeted to specific pro-inflammatory signal-transduction cascades and cytokines potentially applicable to gout treatment is rapidly advancing. However, despite significant advances in understanding and exciting developments of treatments, the management of gout remains sub-optimal due to the undesirable side effects such as gastrointestinal toxicity, bleeding, diarrhoea, and cardiovascular events [9,10]. As a result, there is an urgent need to develop new safe anti-inflammatory treatments with maximum efficacy for gouty arthritis therapy.

**Fig 1:**
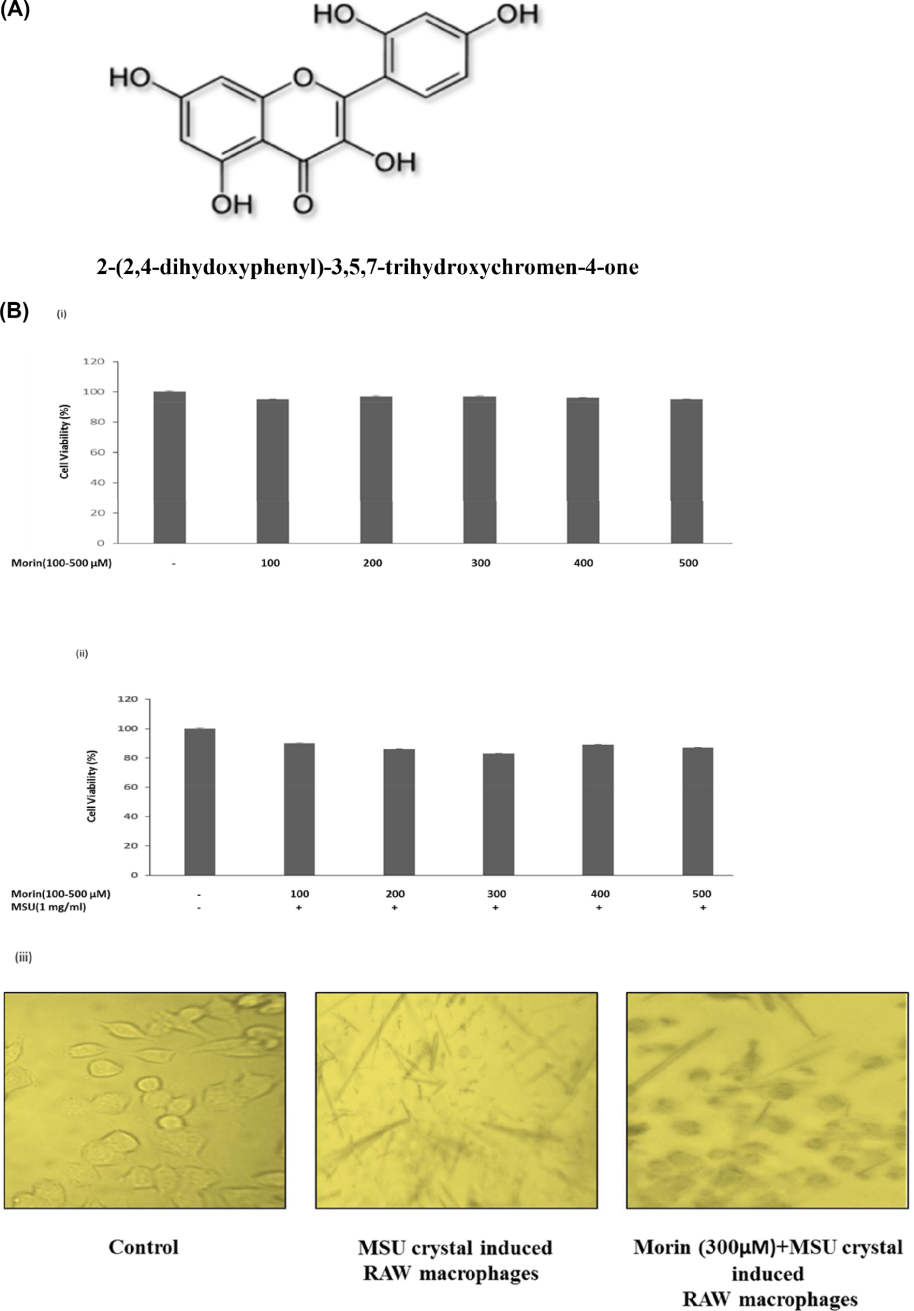
Effect of morin on the cell viability of RAW 264.7 macrophage cells. (a) Structure of morin (b) Cells were seeded in 96-well plate and treated with varying concentration of (i) morin alone (100–500 μM) for 24 h. As well as, (ii) RAW 264.7 macrophage cells were pre-treated with varying concentration of morin (100–500 μM) followed by stimulation with MSU crystal (1 mg/ml) for 24 h. (iii) Cell phenotypes of control, MSU crystal induced and morin+MSU crystal induced RAW macrophages after 24 h were observed under phase-contrast microscope. The results are expressed as mean ±SD of the data from three experiments.

Recently, agents obtained from plants have received increased interest in the treatment of arthritis. The flavonoids are a class of secondary metabolites which has been found in a variety of fruits, juices, vegetables and components of herbal containing dietary supplements [11]. Morin (Fig 1A), a member of natural flavonols, is a yellowish pigment which is isolated from Chinese herbs of the Moraceae family, such as mulberry figs, mill (*Prunus dulcis*), almond hulls, and osage orange (*Maclura pomifera*) [12,13]. Morin has been shown to exhibit many biological activities including antioxidant, cytoprotection, antimutagenesis, antidiabetic, anti-inflammatory and anticarcinogenic effects [14]. It also modulates the transcription factor NF-κB activation through the ERK and p38 MAPKs signalling pathways by its reactive oxygen species scavenging activity [11]. Morin has also been shown to increase the excretion of urate in the urine and decrease the plasma urate level in experimental animals [15,16]. A number of *in vitro* and animal studies have proven the anti-inflammatory effect of morin on lipoxygenase-1, inducible nitric oxide synthase, inflammatory cytokines and cyclo-oxygenase expression in activated immune cells like macrophages and mast cells [17,18]. The recent studies of our laboratory also demonstrated the anti-inflammatory effects of morin against adjuvant-induced arthritic rats [19]. However, no report has been issued on its anti-inflammatory activities against MSU crystal-induced inflammation, Therefore, as part of our ongoing screening program to evaluate the anti-inflammatory properties of morin, we investigated the suppressive effect of morin and its underlying molecular mechanisms against inflammatory immune responses in MSU crystal-induced RAW 264.7 macrophage cells.

## Materials and Methods

### Chemicals

Morin (3,5,7,2’,4’- pentahydroxyflavone), colchicine and TRIzol were purchased from Sigma Chemical Co. (St.Louis, MO, USA). Dulbecco’s modified Eagle’s medium (DMEM), fetal bovine serum (FBS), antibiotics (penicillin and streptomycin) were obtained from Life Technologies Inc. (Grand Island, NY, USA). High Capacity cDNA reverse transcription kit and QuantiTect SYBR Green PCR kit were purchased from Qiagen, (Valencia, CA, USA) and Applied Biosystems (Foster City, CA, USA) respectively for real time-PCR experiments. The primary antibodies against NF-κBp65 (Cell Signaling Technology; Cat No. 4764), COX-2 (Cell Signaling Technology; Cat No. 12282), TNF-α (Cell Signaling Technology; Cat No. 11948), β-actin (Cell Signaling Technology, Beverly, MA, USA; Cat No. 8457) and Lamin A were purchased from Abcam, (Cambridge, UK; Cat No. ab26300). Secondary horseradish peroxidase (HRP)-conjugated antibody was also purchased from Cell Signaling Technology. All other chemicals and reagents were used of analytical grade obtained from commercial sources.

### Monosodium Urate Crystal Synthesis

Briefly, 4 g of uric acid was dissolved and heated in 800 ml H_2_O with NaOH (9 mL/0.5 N), adjusted to pH 8.9 at 60°C, the solution was gradually cooled by stirring at room temperature and stored overnight at 4°C with some modification [20]. The crystals that formed were sterilized by heating at 180°C for 2 h and suspended in sterile saline (20 mg/ml). The crystals obtained by this method were of comparable size (5 to 25 μM long) and needle-shaped, negatively birefringent crystals observed by compensated polarized light microscopy. The bacterial endotoxin levels in MSU crystal preparations were assessed by Limulus amoebocyte cell lysate assay (Pyrogent®-5000 Biowhittaker, Inc., USA).

### Cell Culture and Treatment

RAW 264.7 murine macrophages were purchased from National Centre for Cell Sciences, Pune, India [21]. The cells were maintained in Dulbecco’s modified eagle’s medium (DMEM) supplemented with 10% fetal bovine serum (FBS), 100 U/ml penicillin and 100 μg/ml streptomycin in a humidified atmosphere of 5% CO_2_ at 37°C. Cells were allowed to grow until it reaches 90–95% confluence, and it was washed with phosphate buffered saline and the culture medium was replaced. Cells attaining a concentration of 5x10^4^ cells/ml were activated by incubation in medium containing MSU crystals (1mg/ml). Various concentrations of morin (100–300 μM) were dissolved in DMSO at the respective stock concentration were added directly to cell culture medium and final concentration of DMSO in culture medium was maintained at 0.01% (vol/vol).

### Cell Viability

Cell viability was assessed by 3-(4, 5-dimethylthiazol-2-yl)-2,5-diphenyl tetrazolium bromide (MTT) assay. In brief, RAW 264.7 macrophages were seeded in 96-well plates at a density of 5x10^3^ cells per well. After 24 h, the cells were stimulated with various concentrations of morin (100, 200, 300, 400, 500 μM) with or without MSU crystals (1 mg/ml) for further 24 h. After the incubation, the medium was decanted, and 20 μl of MTT dye solution (5 mg/ml in PBS buffer) were added to each well and the plate was incubated for 4 h at 37°C, Then, 200 μl of DMSO was added to the wells and incubated again for 15 min under gentle shaking at 37°C to dissolve the tetrazolium dye. Relative cell viability was calculated by determining the absorbance at 570 nm and untreated control cells were assigned as a relative viability of %.

### Determination of Lysosomal Enzymes

RAW 264.7 macrophages were seeded in 6 well plates (50,000 cells/well) and cultured overnight prior to incubation. The cells were then treated with morin (100–300 μM) or colchicine (1 μM) prior to the stimulation of MSU crystal (1 mg/ml) for 24 h. At the end of incubation, cells were washed with PBS and then lysed by adding ice cold lysis buffer. The cell lysate from macrophages were prepared by centrifugation at 10,000 for 10 min at 4°C. The cell free supernatant was stored at -80°C until its usage in the assay. Control experiments were performed by measuring the release of enzymes tested in the untreated group. The cell free supernatant was assayed for lysosomal enzyme activity. Acid phosphatase was assayed by the method of King [22] using disodium phenyl phosphate as the substrate. The enzyme activity was expressed as μmoles of phenol liberated/min/mg protein. The activity of β-galactosidase was assessed by the method of Rosenblit et al [23] using 4-nitrophenyl-N-acetyl galactopyranoside as the substrate and its activity was expressed as μmoles of p-nitrophenol liberated/h/mg protein. N-Acetyl glucosaminidase activity was assessed by the method of Maruhn [24] using 4-nitrophenyl-N-acetyl glucosaminide as the substrate, and its activity was expressed as μmoles of p-nitrophenol formed/h/mg of protein. Cathepsin D activity was assayed by the method of Etherington [25] using hemoglobin as the substrate and the activity was expressed as μmoles of tyrosine liberated/h/mg of protein. Protein content was measured by the method of Lowry et al [26].

### Estimation of Lipid Peroxidation

RAW 264.7 macrophages were cultured in 6-well plate with serum free medium and pretreated with different concentrations of morin (100–300 μM) or colchicine (1 μM) for 24 h. The production of lipid peroxidation (LPO) was stimulated by the addition of MSU crystals (1 mg/ml) and incubated for 24 h. The LPO level in cell lysate was estimated by Ohkawa et al [27] method using the thiobarbituric acid and the optical density was measured spectrophotometrically at 532 nm. The values are expressed as nmol of MDA released /mg of protein.

### Determination of Anti-Oxidant Status

Antioxidant status of RAW 264.7 macrophages was determined by estimating the activity of various antioxidant enzymes and levels of non-enzymic cellular antioxidants. The macrophages were initially cultured in 6 well plate and pretreated with different concentrations of morin (100–300 μM) or colchicine (1 μM), incubated for 24 h and cells were stimulated with MSU crystals (1 mg/ml) for 24 h. The super oxide dismutase assay was carried out by the method of Marklund & Marklund [28]. The enzyme activity is expressed as units/mg protein. Catalase assay was carried out using the method proposed by Sinha [29]. The activity of catalase was expressed as μg of H_2_O_2_ consumed/min/mg/protein. The activity of glutathione peroxidase was estimated by the non-enzymatic assay of Rotruck et al [30] and the unit was expressed as μg of glutathione utilized/min/mg/protein. The activity of reduced glutathione was estimated by the method of Ellman [31] and the unit was expressed as nmol/mg protein.

### Measurement of Nitric Oxide Production

The nitrite levels in the culture medium were assessed by Griess reaction. RAW 264.7 macrophage cells (1.5x10^6^ cells/well) were cultured in 6-well plate and pretreated with different concentrations of morin (100–300 μM) or colchicine (1 μM) for 24 h. Cellular NO production was stimulated by adding MSU crystals (1mg/ml), followed by incubation for 24 h. The conditioned medium (100 μl) was then mixed with the same volume of Griess reagent and incubated for 15 min. The absorbance of the mixture at 540 nm was measured with an ELISA microplate reader (Biotek, Winooski, USA). The values were compared with those from standard concentrations of sodium nitrite and then levels of nitrite in the conditioned media of treated cells were calculated.

### Determination of Prostaglandin E_2_ (PGE_2_) Concentration

RAW 264.7 macrophage cells (1.5x10^6^ cells/well) were cultured in 6 well plate with serum free medium and pretreated with different concentrations of morin (100–300 μM) or colchicine (1 μM) for 24 h. The production of PGE_2_ was stimulated by MSU crystals (1 mg/ml) and incubated for 24 h. The levels of PEG_2_ were determined in conditioned medium with the PGE_2_ ELISA kit (Cayman Chemical Co., Ann Arbor, Michigan, USA) according to the manufacturer’s instructions. Absorbance was measured at 450 nm with an ELISA microplate reader (Biotek, Winooski, USA).

### ELISA for Cytokine Determination

The generation of inflammatory gene products (TNF-α, IL-1β, IL-6, MCP-1, and VEGF) by the RAW 264.7 macrophage cells was assayed by ELISA kits (Peprotech, Rocky Hill, NJ) as per the manufacturer’s instructions. Cells (1.5x10^6^ cells/well) in 6-well plates were pre-incubated with various concentrations of morin (100–300 μM) or colchicine (1 μM) for 24 h, and cytokine productions were stimulated by treating the cells with MSU crystals (1 mg/ml) for 24 h. Then, the cultured supernatants were collected and used for measuring TNF-α, IL-1β, IL-6, MCP-1 and VEGF levels. Absorbance was determined at 450 nm using the microplate reader (Biotek, Winooski, USA). The standard curves for respective cytokines were used to quantify the TNF-α, IL-1β, IL-6, MCP-1, and VEGF released by the cells.

### Quantitative Real-Time Polymerase Chain Reaction (RT-PCR) Analysis

Total RNA was isolated from RAW 264.7 macrophage cells after pre-treatment with various concentrations of morin (100–300 μM) or colchicine (1 μM) and then cells were stimulated with MSU (1 mg/ml) using the TRIzol Reagent (Sigma) according to the manufacturer’s instructions. The 2 μg RNA was reverse transcribed by using the higher capacity cDNA reverse transcription kit (Applied Biosystems, CA, USA) and the mRNA expression was amplified by Quantitect SYBR^®^ PCR kit (QIAGEN, Valencia, CA, USA). Gene specific primers were designed manually by using NCBI/primer-BLAST tool software and were purchased from Sigma Aldrich, USA (Table 1). Quantitative RT-PCR (qRT-PCR) was performed to measure TNF-α, IL-1β, IL-6, MCP-1, iNOS, COX-2, NF-κBp65 and HPRT respectively. Transcription levels were assessed utilizing the step one real-time thermal cycler with SYBR Green PCR Master Mix according to the instructions of the manufacturer (Applied Biosystems, Foster City, CA, USA). Amplification was performed in the following cycling conditions: 94°C for 15s, 60°C annealing for 30s, and 72°C extension for 30s. The fold change in gene expression levels of target genes were calculated with normalization to β-actin values using the 2^−ΔΔCt^ comparative cycle threshold method.

**Table 1:** Primer sequences used for quantitative real-time PCR analysis of RNA.

Gene	Primer sequences
TNF-α	F- 5’TGGAACTGGCAGAAGAGGCACT 3’
	R- 5’AGAGGCTGAGACATAGGCACCG 3’
IL-1 β	F- 5’ACCTGGGCTGTCCTGATGAGAG 3’
	R- 5’TGTTGATGTGCTGCTGCGAGAT 3’
IL-6	F- 5’-GTTCTCTGGGAAATCGTGGA 3’
	R- 5’-GGAAATTGGGGTAGGAAGGA 3’
MCP-1	F- 5’AGCACCAGCACCAGCCAACT 3’
	R- 5’GTGAATGAGTAGCAGCAGGTGAGTG 3’
iNOS	F-5’ CAGCGGAGTGACGGCAAACAT 3’
	R- 5’GCAAGACCAGAGGCAGCACATC 3’
COX-2	F-5’ CTGGTGCCTGGTCTGATGATGTATG 3’
	R- 5’TCTCCTATGAGTATGAGTCTGCTGGTT 3’
NFκB	F- 5’GCTCCTAAGGTGCTGACA 3’
	R- 5’ACCTCCGAAAGCGAGATA 3’
HPRT	5’-CCTGCTGGATTACATTAAAGCACTG 3’
	5’-GTCAAGGGCATATCCAACAACAAAC 3’
GAPDH	F- 5’-ACTCCACTCACGGCAAATTC 3’
	R- 5’-GTCATGAGCCCTTCCACAAT 3’

### Western Blotting Analysis

RAW 264.7 macrophage cells were pre-treated with the various concentrations of morin (100–300 μM) or colchicine (1 μM) for 24 h or BAY 11–7082 (10 μM) 1 h prior to the stimulation of MSU crystals (1mg/ml) for the next 24 h. Cytosolic and nuclear extracts were prepared with NE-PER nuclear and cytosolic extraction reagent kit (Thermo-Fisher Scientific, Waltham, Ma, USA) for the detection of NF-κBp65 protein expression,. The whole cell lysate was prepared in ice-cold RIPA buffer mixed with protease inhibitors for the detection of TNF-α and COX-2 protein expression. Protein content was measured by Bradford protein assay kit (Bio-Rad Laboratories Ltd., Mississauga, Canada). Cell lysate (30 μg protein/lane) were resolved by 12% sodium dodecyl sulfate-polyacrylamide gel electrophoresis (SDS-PAGE). Proteins were then transferred onto polyvinylidene fluoride membranes (PVDF) and then blots were blocked overnight at 4°C within 5% (w/v) BSA. The membranes were incubated with the desired primary polyclonal antibody against NF-κBp65 (1:1000); COX-2 (1:1000); TNF-α (1:1000) and β-actin (1:2000) and lamin A (1:1000) overnight at 4°C. After then washed with Tris-buffered saline solution containing 0.1% Tween 20 (TBST) and probed for 1 h with the appropriate secondary antibody (HRP-conjugated goat anti rabbit IgG antibody) (1:10000). Protein bands were visualized using the enhanced chemiluminescence detection system (Bio-Rad Laboratories, Hercules, CA, USA). The densities of the bands were measured using the Bio-RAD Chem DOC^TM^ XRS luminescent image analyzer and image lab version 2.0.1 software system (Bio-Rad, USA).

### Flow Cytometric Analysis of Intracellular ROS Levels

The level of intracellular ROS production was directly measured by using the dye DCFH-DA as described earlier. DCFH-DA, a stable, non-fluorescent, cell permeable compound enters living cells and was deacetylated intracellularly by non-specific esterase, which was further oxidized by reacting with ROS yielding fluorescent compound 2.7-dichlorofluorescein (DCF). Thus, the fluorescence intensity of the probe is indicative of ROS levels within the cells. The cells were incubated with morin (100–300 μM) or colchicine (1 μM) for 24 h and then with or without MSU crystals (1mg/ml) for 24 h. After incubation, cells suspended in phosphate buffered saline (PBS) were stained with 10 μM DCFH-DA at 37°C for 30 min. Next, the cells were plated on ice, washed twice with cold PBS and analyzed by flow cytometry (Becton-Dickinson, San Diego, CA, USA). A minimum of 10,000 events/sample was acquired. Data were reported out in terms of fluorescence intensity units (FIU).

### Statistical Analysis

Results were expressed as mean ± S.D and statistical analysis was performed by GraphPad InStat 3 software, using one-way ANOVA to determine the significant differences between groups, followed by Student’s Newman-Keul’s test. *P<0.05 implied significance.

## Results

### Effect of Morin on the Viability of RAW 264.7 Macrophage Cells

The cytotoxicity of morin to RAW 264.7 macrophage cells were measured by MTT assay. As shown in Fig 1B, cell viability was not significantly altered by 24 h treatment with morin at up to the concentration of 500 μM. This result indicated that concentration of morin below 500 μM was not toxic to RAW 264.7 macrophage cells. Therefore, in the subsequent experiments, morin was used at a concentration range of 100–300.

### Effect of Morin on Lysosomal Enzymes in MSU Crystal Stimulated RAW 264.7 Macrophage Cells

To assess the effect of morin on the release of lysosomal enzymes (acid phosphatase, β-galactosidase, N-Acetyl glucosaminidase and cathepsin D) in MSU crystal stimulated macrophages, cells were treated with or without morin (100–300 μM) or colchicine (1 μM) prior to the stimulation of MSU crystals (1 mg/ml) for 24 h. The cell free supernatant was used for assaying for lysosomal enzyme activity. As shown in Table 2, a significant increase in the level of lysosomal enzyme activities was observed in the MSU crystal stimulated RAW 264.7 macrophage cells. However, we found that morin treatment significantly decreased the levels of lysosomal enzyme activities similar to that of colchicine in MSU crystal stimulated RAW 264.7 macrophage cells.

**Table 2:** Effect of morin on MSU crystal-induced release of lysosomal acid hydrolases in RAW 264.7 macrophage cells.

Parameter	Control	MSU (1mg/ml)	MSU+Morin (100μM)	MSU+Morin (200μM)	MSU+Morin (300μM)	MSU+Colchicine (1μM)
**Cell lysate**						
β-galactosidase	2.46±0.03	3.24±0.04^a*^	2.81±0.05^a*b*^	2.58±0.03^a*b*c*^	2.2±0.22^a*b*c*d*^	2.6±0.13^b*e*^
N-acetyl-β-D- glucasaminidase	3.63±0.02	5.2±0.02^a*^	4.42±0.02^a*b*^	4.03±0.02^a*b*c*^	3.9±0.10^a*b*c*^	4.3±0.13^a*b*d*^
Cathepsin D	3.95±0.03	9.28±0.04^a*^	7.97±0.01^a*b*^	7.36±0.02^a*b*^	7.2±0.08^a*b*c*^	8.3±0.13^a*b* e*^
Acid Phosphatase	4.16±0.1	6.33±0.05^a*^	5.64±0.04^a*b*^	5.44±0.03^a*b*c*^	5.44±0.03^a*b*c*^	5.4±0.13^a*b*e*^
**Supernatant**						
β-galactosidase	3.26±0.05	6.53±0.06^a*^	4.56±0.05^b*^	4.15±0.02^a*b*^	3.98±0.14^a*c*d*^	4.06±0.12^b*d*e*^
N-acetyl-β-D- glucasaminidase	4.87±0.05	7.92±0.01^a*^	5.23±0.01^a*b*^	4.96±0.04^a*b*c*^	4.03±0.18^a*b*c*^	4.51±0.14^a*d*e*^
Cathepsin D	5.26±0.05	10.5±0.02^a*^	9.34±0.03^a*^	8.74±0.05^a*b*c*^	8.37±0.05^b*c*d*^	8.25±0.01^a*d*e*^
Acid Phosphatase	6.75±0.4	9.86±0.04^a*^	7.72±0.06^a*b*^	7.01±0.06^a*b*c*^	6.89±0.03^a*c*d*^	7.2±0.04^a*c*d*^

RAW 264.7 cells were pre-treated with various concentrations of morin (100–300 μM) and then stimulated with MSU crystal (1 mg/ml) for 24 h. Enzyme activities are expressed as: β-galactosidase and N-acetyl glucosaminidase-micromoles x10^-2^ of p-nitrophenol liberated/h/milligram protein; Cathepsin D-micromoles x10^-2^ of tyrosine liberated/h/milligram protein; acid phosphatase-micromoles x10^-2^ of phenol. The results are expressed as means ±SD of the data from three experiments. Comparisons are made with: a—Control versus MSU crystal-induced RAW 264.7 macrophage cells, MSU+morin-treated RAW 264.7 macrophage cells (100–300 μM) and MSU+colchicine (1 μM) treated macrophage cells; b—MSU crystal-induced RAW 264.7 macrophage cells versus MSU+morin-treated RAW 264.7 macrophage cells (100–300 μM) and MSU+colchicine (1μM) treated RAW 264.7 macrophage.

### Effect of Morin on Lipid Peroxidation and Anti-Oxidant Status in MSU Crystal Stimulated RAW 264.7 Macrophage Cells

To investigate the effect of morin on lipid peroxidation and anti-oxidant status in MSU crystal-stimulated RAW 264.7 macrophage cells, cells were treated with or without morin (100–300 μM) or colchicine (1 μM) prior to the stimulation of MSU crystals (1 mg/ml) for 24 h. As depicted in Table 3, MSU crystals stimulation dramatically enhanced the production of LPO and reduced the anti-oxidant status (superoxide dismutase, catalase, glutathione peroxidase, and reduced glutathione) in RAW 264.7 macrophage cells. On the other hand, treatment with morin showed a marked decrease in the elevated LPO level in association with increased anti-oxidant status similar to that of colchicine when compared with untreated specimen.

**Table 3:** Effect of morin on MSU crystal-induced lipid peroxidation and anti-oxidant status in RAW 264.7 macrophage cells.

Parameter	Control	MSU (1mg/ml)	MSU+Morin (100μM)	MSU+Morin (200μM)	MSU+Morin (300μM)	MSU+Colchicine (1μM)
**Lipid peroxidation**	4.38±0.05	4.80+0.1^a*^	4.25±0.08^b*^	3.9±0.03^a*b*^	2.24±0.02^a*b*c*^	4.0±0.04^a*b*e*^
**SOD**	2.6±0.06	1.55±0.01^a*^	1.93±0.04^a*b*^	2.14±0.08^b*^	2.6±0.08^b*c*d*^	2.2±0.2^a*b*c*^
**CAT**	5.39±0.26	2.9±0.01^a*^	4.74±0.08^a*b*^	5.16±0.26^b*c*^	5.19±0.14^b*c*^	5.17±0.013^b*c*^
**GPx**	15.94±0.8	6.7±0.7^a*^	14±0.8^b*^	14.98±1.0^b*c*^	17.2±0.79^a*b*c*d*^	13.5±0.8^a*b*e*^
**GSH**	0.77±0.01	0.28±0.06^a*^	0.46±0.08^a*b*^	0.57±0.08^a*b*c*^	0.62±0.08^b*c*^	0.69±0.01^b*c*d*^

RAW 264.7 cells were pre-treated with various concentration of morin (100–300 μM) and then stimulated with MSU crystal (1 mg/ml) for 24 h. Enzyme units are expressed as; Lipid peroxidation-nm of malondialdehyde formed/mg protein; Superoxide dismustase (SOD)—units/mg protein(unit amount of enzyme required to inhibit the auto-oxidative reaction by 50%); Catalse (CAT)—μmol of H_2_O_2_ consumed/min/mg protein; Glutathione peroxidase (GPx)—μg of GSH utilized/min/mg protein and Reduced glutathione (GSH)—mg/100 g. Comparisons are made with: a—Control versus MSU crystal-induced RAW 264.7 macrophage cells, MSU+morin-treated RAW 264.7 macrophage cells (100–300 μM) and MSU+colchicine (1 μM) treated macrophage cells; b- MSU crystal-induced RAW 264.7 macrophage cells versus MSU+morin-treated RAW 264.7 macrophage cells (100–300 μM) and MSU+colchicine (1 μM) treated RAW 264.7 macrophage cells; c,d,e—MSU+morin treated RAW 264.7 macrophage cells (100–300 μM) versus MSU+colchicine (1 μM) treated RAW 264.7 macrophage cells. *P<0.05 statistically significant.

### Effect of Morin on Pro-Inflammatory Mediators in MSU Crystal Stimulated RAW 264.7 Macrophage Cells

**Fig 2:**
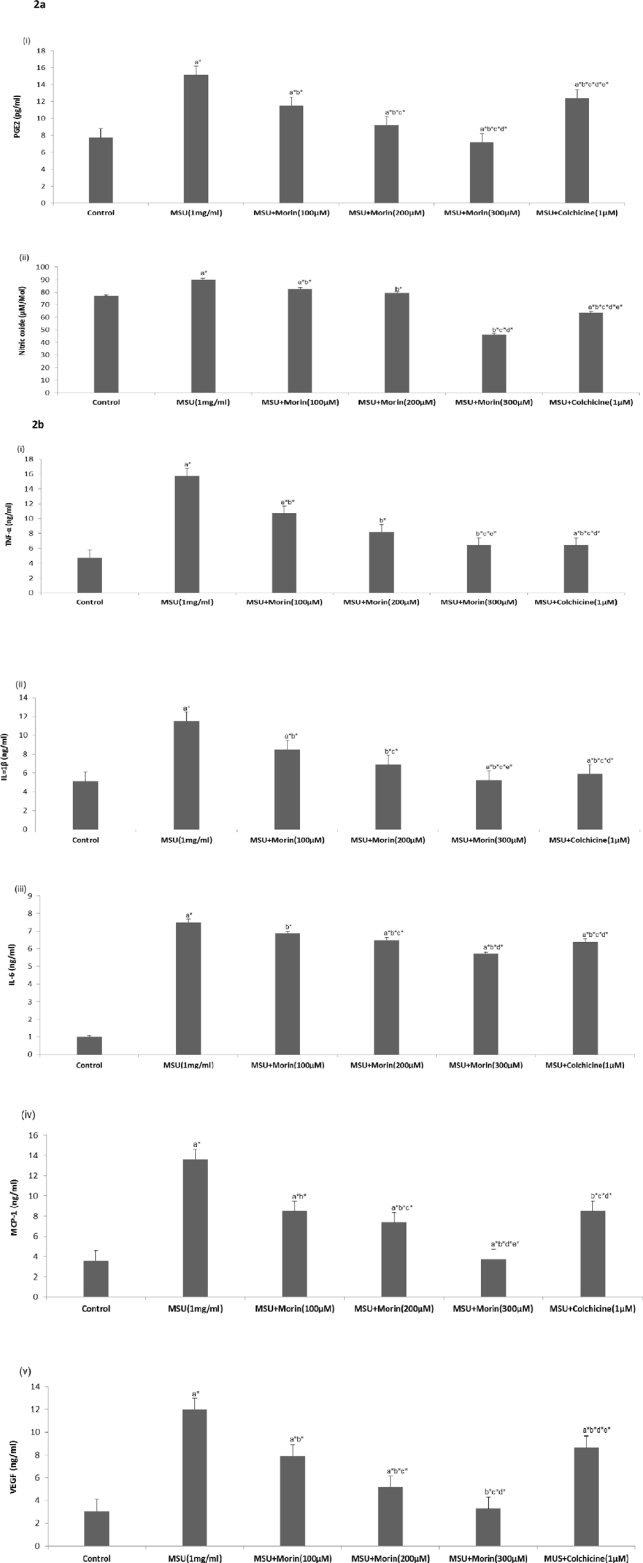
Effect of morin on MSU crystal-induced production of pro-inflammatory meditors in RAW 264.7 macrophage cells. (a) The levels of (i) PGE_2_ and NO were measured in the culture medium. (b) The level of (i) TNF-α, (ii) IL-1β, (iii) IL-6, (iv) MCP-1, (v) VEGF and PGE_2_ in the culture medium was measured by its respective ELISA kits. The results are expressed as means ±SD of the data from three experiments. Comparisons are made with: a—Control versus MSU crystal-induced RAW 264.7 macrophage cells, MSU+morin-treated RAW 264.7 macrophage cells (100–300 μM) and MSU+colchicine (1 μM) treated macrophage cells; b—MSU crystal-induced RAW 264.7 macrophage cells versus MSU+morin-treated RAW 264.7 macrophage cells (100–300 μM) and MSU+colchicine (1 μM) treated RAW 264.7 macrophage cells; c, d, e—MSU+morin treated RAW 264.7 macrophage cells (100–300 μM) versus MSU+colchicine (1μM) treated RAW 264.7 macrophage cells. *P<0.05 statistically significant.

To ensure whether morin inhibits the production of PEG_2_ and NO in MSU crystal stimulated RAW 264.7 macrophage cells, the cells were treated with or without morin (100–300 μM) or colchicine (1 μM) prior to the stimulation of MSU crystals (1 mg/ml). As represented in Fig 2A, MSU crystals stimulation was found to promote significantly the production of NO and PEG_2_ in the RAW 264.7 macrophage cells. However, treatment with morin showed significant suppression in the production of PEG_2_ and NO when compared to untreated cells.

As shown in Fig 2B, under basal conditions, macrophages produced low levels of pro-inflammatory cytokines (TNF-α, IL-1β, IL-6, MCP-1, and VEGF), while stimulation with MSU crystals induced a high secretion. However, morin treatment (100–300 μM) significantly inhibited MSU crystal-induced secretion of pro-inflammatory cytokines (TNF-α, IL-1β, IL-6, MCP-1, and VEGF) similar to that of colchicine in RAW 264.7 macrophage cells.

### Effect of Morin on mRNA Expression of TNF-α, IL-1β, IL-6, MCP-1, iNOS, COX-2, NF-κBp65 and HPRT in MSU Crystal Stimulated RAW 264.7 Macrophage Cells

**Fig 3:**
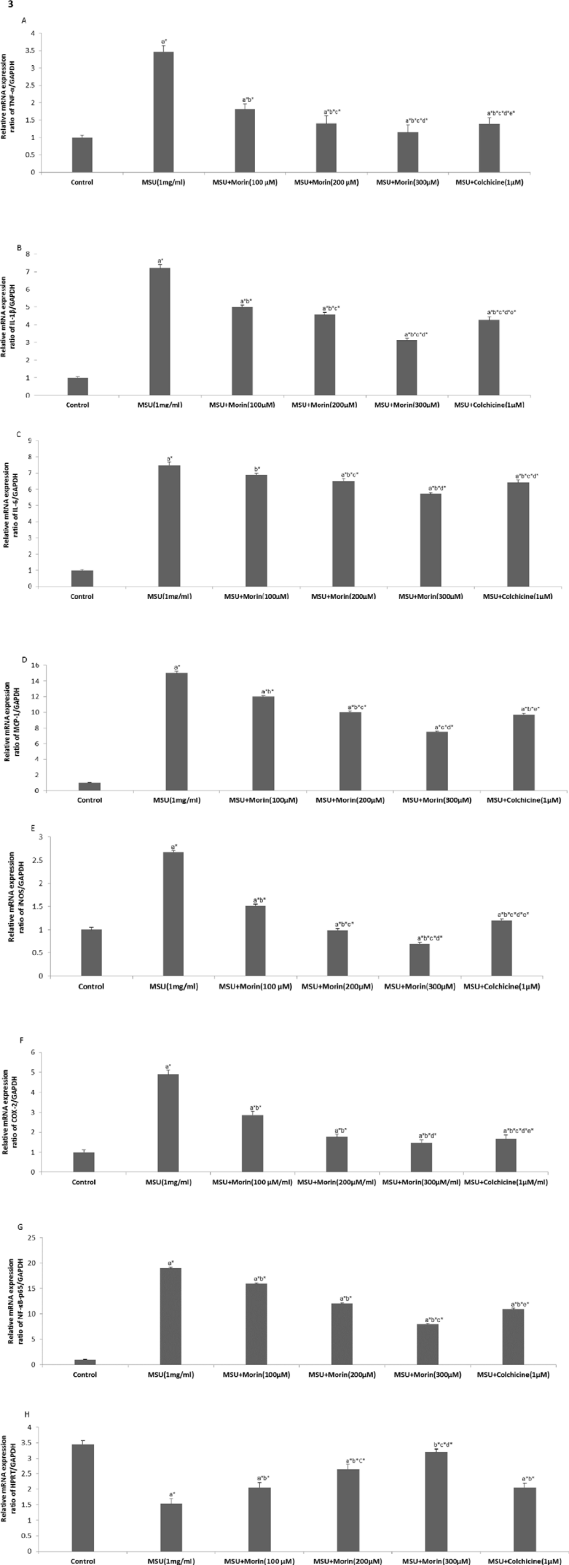
Effect of morin on MSU crystal-induced mRNA expression levels in RAW 264.7 macrophage cells. The mRNA expression of (A) TNF-α, (B) IL-1β, (C) IL-6, (D) MCP-1, (E) iNOS, (F) COX-2, (G) NF-κBp65, (H) HPRT were measured with RT-PCR analysis. RQ values are calculated relative to the GAPDH gene. The results are expressed as mean ±SD of the data from three experiments. Comparisons are made with: a—Control versus MSU crystal-induced RAW 264.7 macrophage cells, MSU + morin treated RAW 264.7 macrophage cells (100–300 μM) and MSU+colchicine (1μM) treated RAW 264.7 macrophage cells; b—MSU crystal-induced RAW 264.7 macrophage cells versus MSU+morin treated RAW 264.7 macrophage cells (100–300 μM and MSU+colchicine (1μM) treated RAW 264.7 macrophage cells; c,d,e—MSU+morin treated RAW 264.7 macrophage cells (100–300 μM) versus MSU+colchicine (1 μM) treated RAW 264.7 macrophage cells. *P<0.05 statistically significant.

To investigate whether morin regulated transcriptional level of pro-inflammatory mediators, cytokines, and HPRT in MSU crystal stimulated RAW 264.7 macrophage cells, cells were treated with the presence or absence of morin (100–300 μM) or colchicine (1μM) prior to the stimulation of MSU crystals (1mg/ml). As shown in Fig 3, MSU crystals stimulation showed up-regulation of NF-κBp65 and NF-κB target inflammatory genes encoding cytokines, (TNF-α, IL-1β and IL-6), chemokine (MCP-1), and inflammatory enzymes (COX-2 and iNOS) in RAW 264.7 macrophage cells. Whereas, the mRNA expression of HPRT was found to be decreased. On the other hand, morin treatment showed down-regulation of the mRNA expression of NF-κBp65 and NF-κB target inflammatory genes in association with the upregulation of HPRT in MSU crystal stimulated RAW 264.7 macrophage cells.

### Effect of Morin on MSU Crystal-Induced NF-κB Signaling Pathway in RAW 264.7 Macrophage Cells

**Fig 4:**
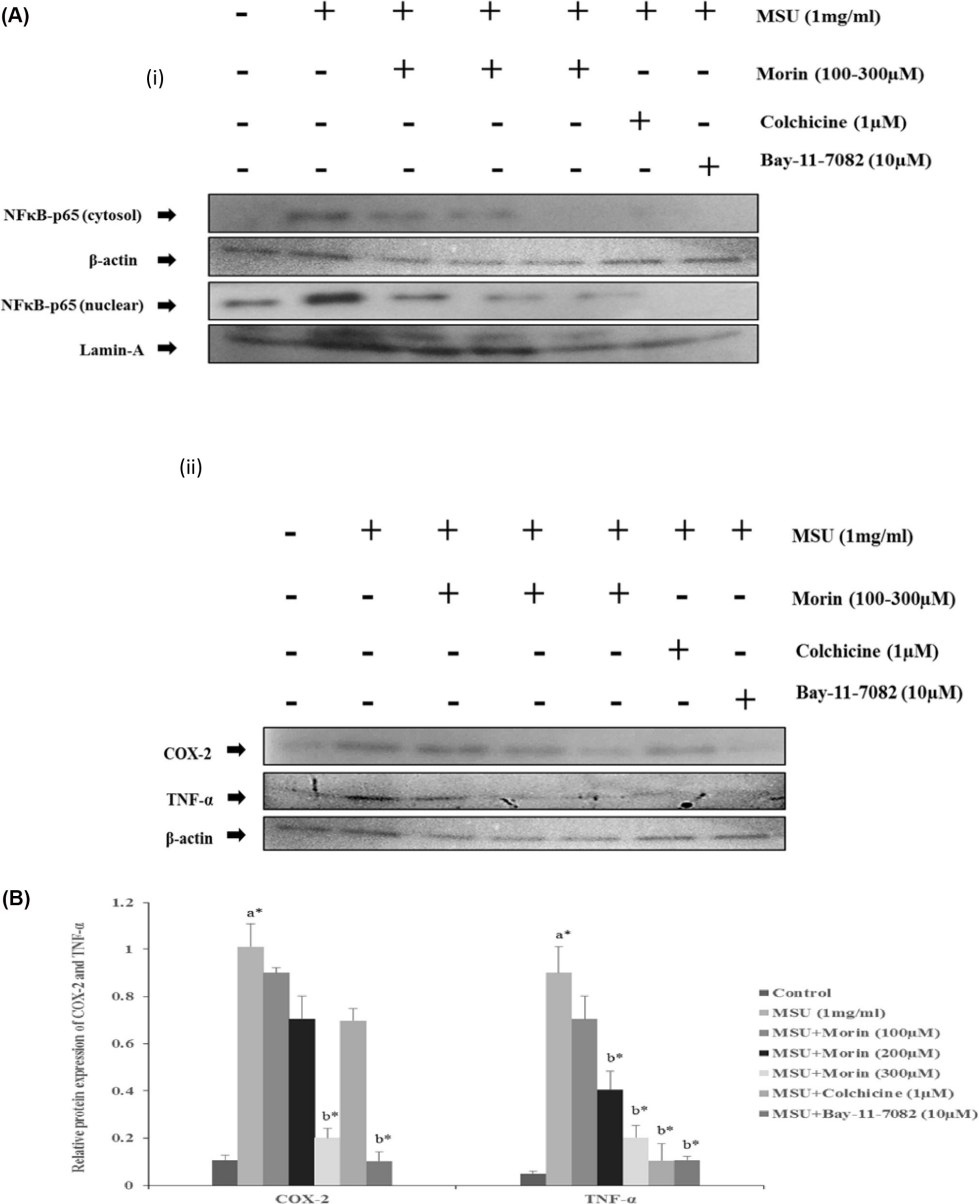
Effect of morin on MSU crystal induced activation of NF-κBp65, COX-2 and TNF-α in RAW 264.7 macrophage cells. RAW 264.7 macrophage cells were treated with varying concentrations of morin (100–300 μM/ml) for 24 h and BAY 11–7028 (10 μM) for 1 h prior to the stimulation of MSU (1 mg/ml) for 24 h. Western blotting analysis was performed as described in materials and methods (i) The expression and translocation of NF-κBp65 protein were evaluated by measuring protein levels in the cytosolic and nuclear cell fractions (ii) COX-2 and TNF-α protein expression were evaluated in the whole cell lysates. β-actin and Lamin A were used as an internal loading control. The values are expressed as mean ± SEM of three independent experiments. Comparisons were made as follows: a—Control versus MSU (1mg/ml) stimulated, MSU+ morin treated groups (100–300 μM), MSU + Colchicine (1 μM); b—MSU stimulated versus MSU + morin (100–300 μM), MSU + Colchicine (1μM). *P<0.05 statistically significant, one-way ANOVA with the Bonferroni multiple comparison post-test.

To confirm whether the inhibitory effects of morin on MSU crystal-stimulated inflammatory mediators (COX-2 and TNF-α) are due to its influence on the activations of NF-κB signaling, we used BAY 11–7082, an IκB kinase inhibitor. RAW 264.7 macrophage cells were pre-treated in the presence or absence of morin (100–300 μM) or colchicine (1μM) for 24 h or BAY 11–7082, an IκB kinase inhibitor (10 μM) for 1 h prior to the stimulation of MSU crystals (1mg/ml). As shown in Fig 4, the western blot analysis clearly showed that morin mainly exerts its anti-inflammatory effects by inhibiting the MSU crystal-induced NF-κBp65 protein expression in cytosolic and nuclear fractions of RAW 264.7 macrophage cells, which subsequently reduced the over expression of COX-2 and TNF-α proteins similar to that of observed in BAY 11–7082.

### Effect of Morin on MSU Crystal Induced Intracellular Free Radical Production

**Fig 5:**
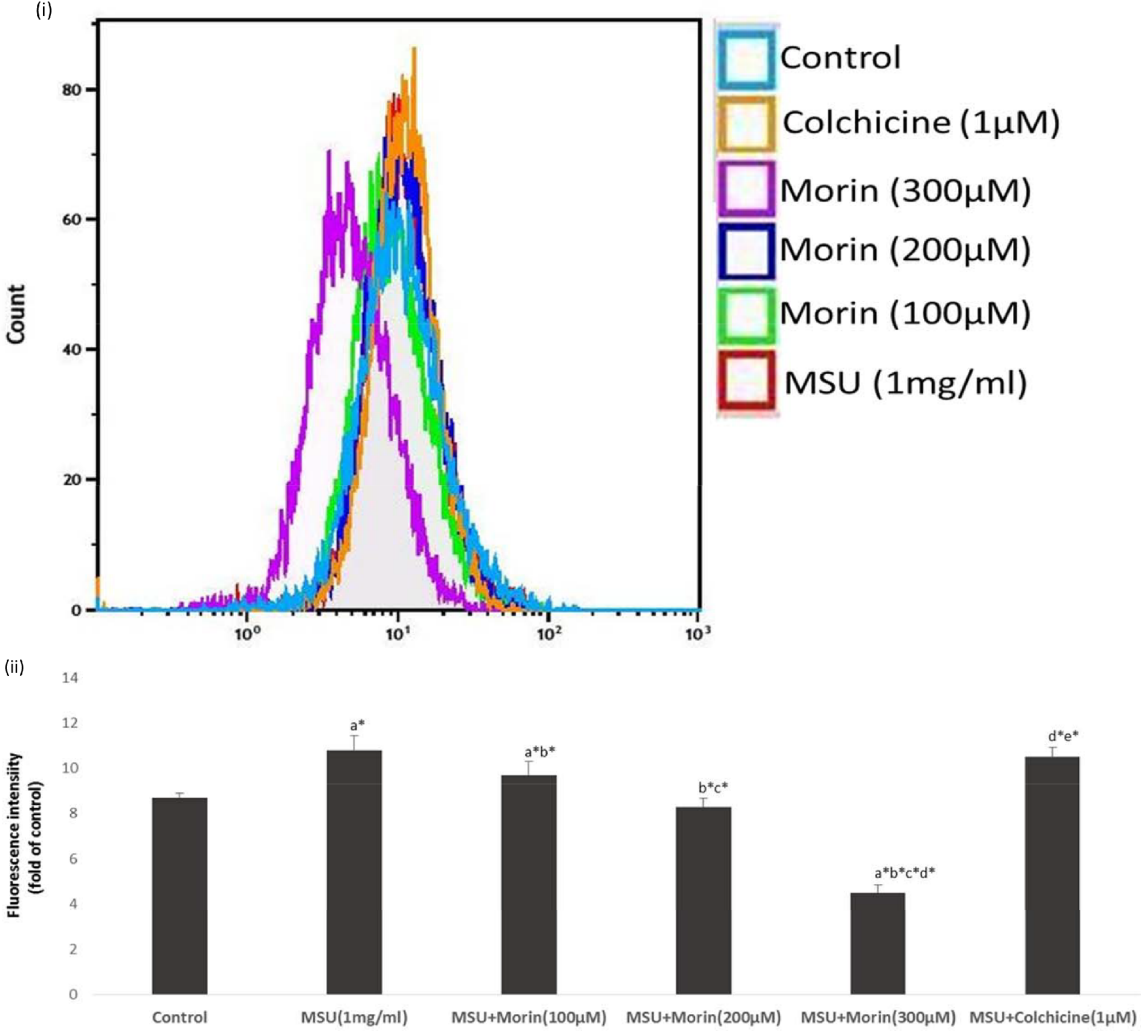
Effect of morin on MSU crystal–induced intracellular ROS generation in RAW 264.7 macrophage cells. (i) Representing the overlay image of flow cytometry. (ii) Data analysis from six experiments performed with different groups and expressed as relative fluorescence (mean ± S.D). Comparisons are made with: a—Control versus MSU crystal-induced RAW 264.7 macrophage cells, MSU+morin treated RAW 264.7 macrophage cells (100–300 μM) and MSU+colchicine (1 μM) treated RAW 264.7 macrophage cells; b—MSU crystal-induced RAW 264.7 macrophage cells versus MSU+morin treated RAW 264.7 macrophage cells (100–300 μM and MSU+colchicine (1μM) treated RAW 264.7 macrophage cells; c, d, e -MSU+morin treated RAW 264.7 macrophage cells (100–300 μM) versus MSU+colchicine (1μM) treated RAW 264.7 macrophage cells. *P<0.05 statistically significant.

Free radicals are the important factors in the inflammatory process as well as in the regulation of NF-κB signaling pathways. So, we investigated the effects of morin on intracellular free radical production in MSU crystal stimulated RAW 264.7 macrophage cells. As shown in Fig 5(i) & 5(ii), MSU crystal stimulation increased the intracellular free radical production in RAW 264.7 macrophage cells compared to control cells. The DCFH-DA fluorescence intensity was about 2.1 fold greater than that of control cells upon exposure to 1mg/ml of MSU crystals. However, treatment of morin (100–300 μM) diminished the MSU crystal-induced free radical increase in RAW 264.7 macrophage cells in a dose dependent manner, with the DCFH-DA fluorescence decreasing by 1.1, 2.5, and 6.3 fold respectively, while colchicine treated MSU crystals stimulated RAW macrophage cells showed DCFH-DA fluorescence decreasing by 0.3 fold.

## Discussion

Our earlier reports from our laboratory confirmed that morin had promising ant-inflammatory and arthritic effects against adjuvant-induced arthritic rats [19]. In order to explore its anti-inflammatory molecular mechanisms against acute gouty arthritis, the present study was designed to investigate the inhibitory effect of morin on the production and expression of pro-inflammatory cytokines, inflammatory enzymes, HPRT, transcription factors and intracellular ROS levels in monosodium urate crystal-induced inflammation in RAW 264.7 macrophage cells, an experimental *in vitro* model for acute gouty arthritis.

Several lines of evidence indicate that MSU crystals are one of the most potent pro-inflammatory stimuli which can initiate, amplify, and sustain an intense inflammatory response in the joint cavity which is intimately responsible for the pathology of gouty arthritis [32,33]. It has been reported that phagocytosis of MSU crystals by macrophages activates the formation of inflammasome, a mutimeric protein complex, which in turn cleaves pro-IL-1β to active IL-1β by activating caspase-1 [34]. IL-1β is the pivotal inflammatory mediator that regulates cell proliferation, differentiation, and apoptosis in gouty arthritis. In addition, IL-1β can induce the expression of a wide range of cytokines and chemokines like TNF-α, IL-6, IL-17, and MCP-1 that are directly responsible for the influx of neutrophils into the synovium, a hallmark of gouty arthritis. Furthermore, MSU crystals stimulate the secretion of TNF-α and IL-6 by synovial cells, monocyte-macrophages, and neutrophils, which induce inflammation [35]. Thus, the blockade of secretion of the inflammatory mediators from MSU crystal activated macrophages could be beneficial for the control and management of acute gouty arthritis. HPRT catalyzes an early step in the salvage pathway for guanine and hypoxanthine metabolism and their deficiency usually leads to hyperuricaemia, precocious gout and uric acid nephrolithiasis [36]. In this study, our results showed that morin markedly decreased the secretion of the cytokines (TNF-α and IL-1β), chemokine (MCP-1), and VEGF in MSU crystal stimulated RAW 264.7 macrophage cells. In addition, the mRNA expression of pro-inflammatory cytokines (TNF-α, IL-1β, IL-6, and IL-17), and chemokine (MCP-1) was also found downregulated in MSU crystal stimulated RAW 264.7 macrophage cells upon morin treatment. Whereas, the HPRT transcriptional level was seen upregulated. It suggested that morin attenuated the inflammatory responses through inhibiting the production of pro-inflammatory mediators.

NO is an inflammatory mediator which is produced by the nitric oxide synthase (NOS). There are three forms of NOS; among these inducible nitric oxide synthase is induced by cytokines for the formation of NO. Nitric oxide (NO) generated by iNOS may react with superoxide anion causing oxidative/nitrosative stress resulting in joint damage [37]. It is well known that the expression of COX-2 is associated with the generation of prostaglandin E_2_ (PGE_2_). Inflammatory signals greatly enhance COX-2 expression, particularly in inflammatory cells such as monocytes and macrophages. PGE_2_ is a potent inflammatory mediator appear to play a prominent role in the onset of gouty attacks by inducing vasodilation and recruitment of neutrophils to the synovial joints [38]. In the present study, we demonstrated that morin treatment suppressed the production of NO and PEG_2_ as well as the expression of iNOS and COX-2 at mRNA level in RAW 264.7 macrophage cells in response to MSU crystals. Since, iNOS and COX-2 are needed for the formation of NO and PEG_2_, therefore, we speculated that the inhibition of NO and PEG_2_ production by morin may be associated with the regulated expression of iNOS and COX-2.

Macrophages stimulated by MSU crystals cause membrane damage that ultimately leads to rupture of lysosomal membrane which results in the release of lysosomal enzymes that can degrade proteins, glycosaminoglycans, nucleic acid and lipids. It has been well established that during inflammation, various cell types, especially the macrophages and neutrophils can directly produce free radicals, which ultimately leads to lipid peroxidation and impair anti-oxidant status through the oxidative stress and generation of reactive oxygen species [20]. Free radicals are a critical determinant that regulates the signaling mechanism leading to the induction of pro-inflammatory cytokines. Free radicals also act as an intracellular second messenger, thereby modulates the downstream transcription factors such as NF-κB, by targeting cysteine and methionine residues on these proteins [39]. Since, the release of free radicals and lysosomal enzymes from the macrophages/monocytes are crucial to the perpetuation of tissue injury and inflammation, the reduction in the release of such enzymes and free radical would be beneficial for the control of acute gouty arthritis. Interestingly, in this study, morin treatment brought a significant decrease in the levels of intracellular free radicals, lipid peroxidation, and lysosomal enzymes in association with an increase in anti-oxidant status in MSU crystals stimulated RAW 264.7 macrophage cells when compared MSU crystals stimulated cells alone.

NF-κB is a transcription factor that acts as a significant part on the onset of inflammation by modulating the expression of pro-inflammatory cytokine (TNF-α, IL-1β, IL-6 and IL-17) genes involved in immune responses, which are all closely connected with the pathogenesis of arthritis. Monocytes/macrophages that encounter with MSU crystals leads to activation of NF-κB that occurs mainly through the phosphorylation and degradation of IκB by IKK. The NF-κB dimer, formerly bound to IκB in the cytosol in its active state, dissociates from IκB and translocates to the nucleus where it induces the expression of various inflammatory genes. The inflammatory gene products further activate transcription factor NF-κB and leads to the expression of inflammatory mediators, including chemokines, matrix metalloproteinases and adhesion molecules, which contributes to the pathogenesis of gouty arthritis. Several reports have demonstrated that activation of NF-κB occurs by phosphorylation of NF-κB proteins such as p65 that results in nuclear translocation [40]. Thus, the drugs that have the capability to inhibit the phosphorylation of NF-κBp65 could block the nuclear translocation of NF-κB. In this present study, we found that morin treatment inhibited the expression of NF-κBp65 at both gene and protein levels in MSU crystals stimulated RAW 264.7 macrophage cells. This result indicates that morin inhibited the nuclear translocation of NF-κB in MSU crystal stimulated RAW 264.7 macrophage cells.

Furthermore, to understand the anti-inflammatory action of morin might be due to the inhibition of the NF-κB pathway, we studied the effect of morin and BAY 11–7082 (IκB kinase inhibitor) on COX-2 and TNF-α production in MSU crystal stimulated RAW 264.7 macrophage cells. As per the western blot analysis, morin mainly exerts its anti-inflammatory effects by inhibiting the MSU crystal-induced COX-2 and TNF-α protein expressions through inactivation of NF-κB signaling pathway in RAW 264.7 macrophage cells similar to that of BAY 11–7082 (IκB kinase inhibitor). The data above indicate that NF-κB is a pivotal transcription factor for inflammatory signaling pathway. Interestingly, our results demonstrate that morin suppress the expression of inflammatory mediators (COX-2 and TNF-α) through the inactivation of NF-κB signaling pathway in MSU crystal stimulated RAW 264.7 macrophage cells.

## Conclusions

In conclusion, morin, a bioflavonoid ameliorated the MSU crystals induced inflammatory response in RAW 264.7 macrophage cells by suppressing the production and expression of pro-inflammatory cytokines, inflammatory enzymes and inflammatory mediators through the inhibition of intracellular ROS levels and NF-κB signaling pathway. Thus, the present study provides a rationale for the use of morin as an anti-arthritic drug. However, future studies using relevant animal models are needed to confirm its anti-arthritic property, which is on underway.

## Supporting Information

S1 Fig Effect of morin on NF-κBp65 protein expression and localization in MSU crystal induced RAW 264.7 macrophages. (TIF)

S2 Fig Effect of morin on COX-2 protein expression in MSU crystal induced RAW 264.7 macrophages. (TIF)

S3 Fig Effect of morin on TNF-α protein expression in MSU crystal induced RAW 264.7 macrophages. (TIF)
