# Supplementary figures and images for: Morin, a Bioflavonoid Suppresses Monosodium Urate Crystal-Induced Inflammatory Immune Response in RAW 264.7 Macrophages through the Inhibition of Inflammatory Mediators, Intracellular ROS Levels and NF-κB Activation
Source: PLoS One. 2015 Dec 28;10(12):e0145093. doi: 10.1371/journal.pone.0145093 (PMC4692533; doi:10.1371/journal.pone.0145093)

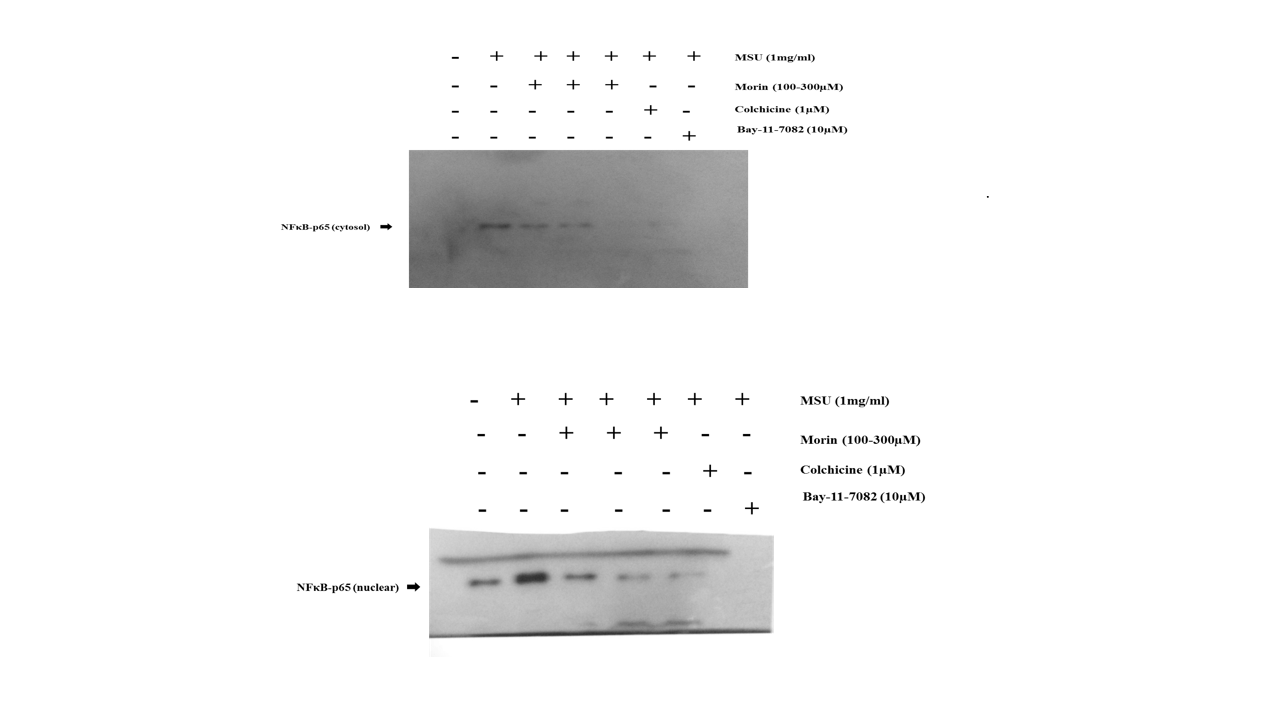

Supplement: S1 Fig — (TIF) [file pone.0145093.s001.tif]

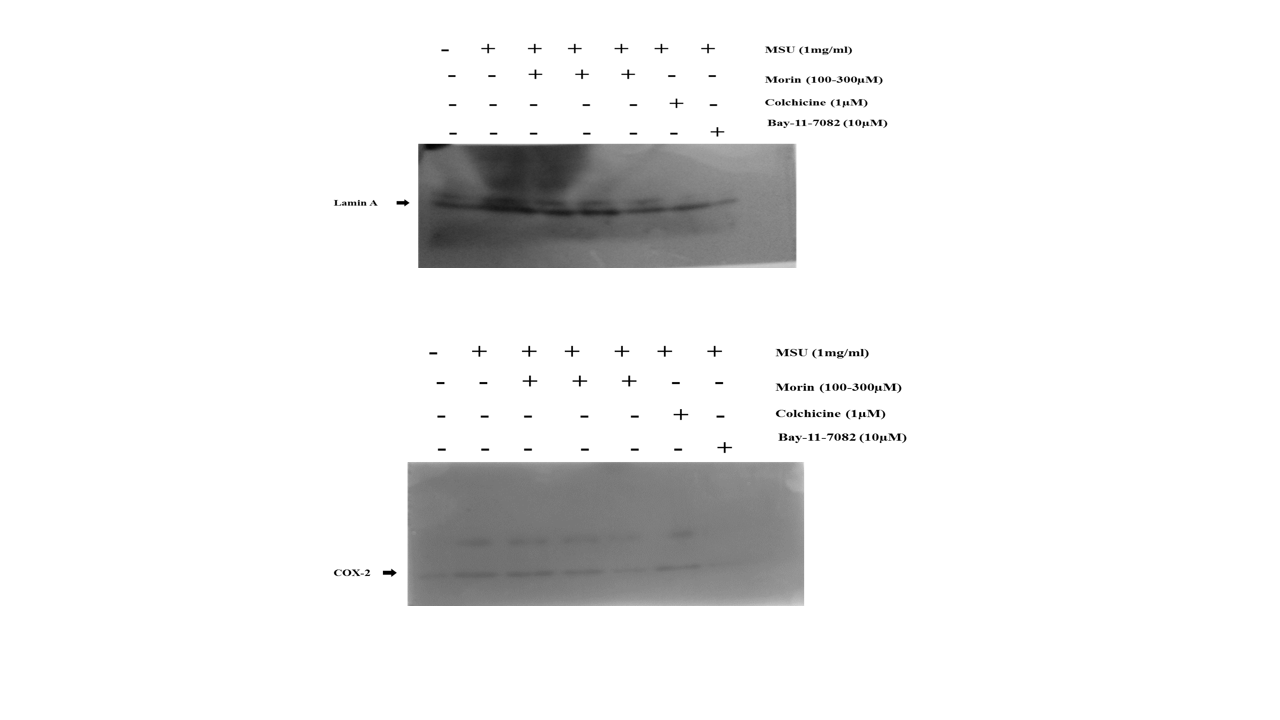

Supplement: S2 Fig — (TIF) [file pone.0145093.s002.tif]

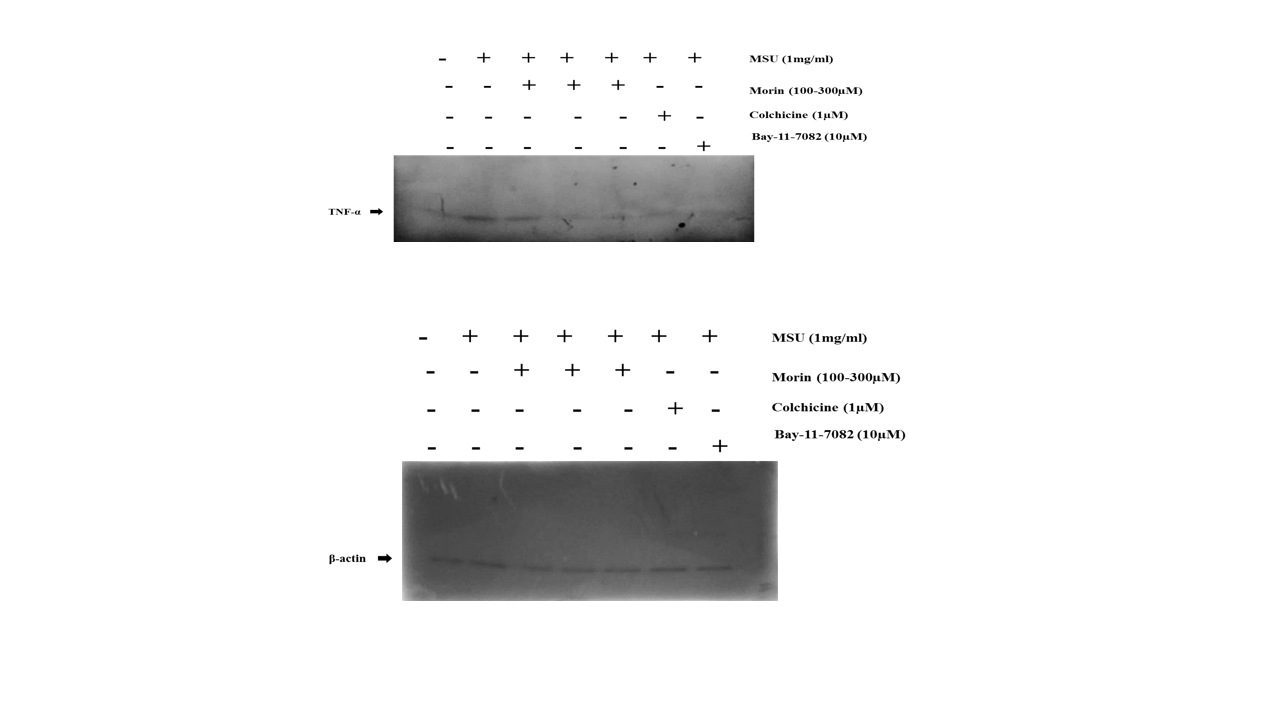

Supplement: S3 Fig — (TIF) [file pone.0145093.s003.tif]
